# Bidirectional Association Between Asthma and Obesity During Childhood and Adolescence: A Systematic Review and Meta-Analysis

**DOI:** 10.3389/fped.2020.576858

**Published:** 2020-10-29

**Authors:** Li-Shen Shan, Qian-Lan Zhou, Yun-Xiao Shang

**Affiliations:** Department of Pediatrics, Shengjing Hospital of China Medical University, Shenyang, China

**Keywords:** obesity, asthma, children, adolescents, cohort studies, meta-analysis 4

## Abstract

**Objective:** Co-occurrence of pediatric asthma and obesity has been widely reported, yet the causal directions between these two disorders are still not well-understood. The objective of this meta-analysis is to explore whether there is a possibility of a bidirectional association for these two disorders in children and adolescents.

**Methods:** PubMed, Embase, Web of Science, and CENTRAL databases were searched up to August 2020. Cohort studies reporting the associations of obesity with risk of physician-diagnosed asthma or physician-diagnosed asthma with risk of obesity in children and adolescents were eligible for the review.

**Results:** A total of 3,091 records were identified from the four databases, with final inclusion of nine. Six studies reported the association between obesity and risk of asthma; three studies reported the association between asthma and risk of childhood obesity. As evaluated by the Newcastle–Ottawa quality assessment scale, all studies were assessed as high-quality studies. There was a statistically significant association between obesity and increased risk of physician-diagnosed asthma in children and adolescents. The pooled RR was 1.39 (95% CI: 1.28, 1.50; *p* < 0.001), with significant heterogeneity across studies (*I*^2^ = 81.7%; *p*_heterogeneity_ < 0.001). The pooled RR in boys was 1.53 (95% CI: 1.17, 1.99; *p* = 0.002), but such a significant association was not observed in girls (RR = 1.17, 95% CI: 0.79, 1.72; *p* = 0.434). For the association of asthma with risk of childhood obesity, the pooled RR was 1.47 (95%CI: 1.25, 1.72; *p* < 0.001) without statistical heterogeneity (*I*^2^ = 0%, *p*_heterogeneity_ = 0.652).

**Conclusion:** There is a bidirectional association between obesity and asthma during childhood and adolescence, suggesting that childhood obesity drives an increase in the onset of asthma; meanwhile, childhood asthma may also increase risk of obesity for children and adolescents.

## Introduction

Pediatric obesity and asthma are both major chronic childhood disorders worldwide in recent decades (1, 2). Increasing epidemiological studies have focused on the concomitant rise in these two disorders, yet the temporal relationship has not been well-established (3, 4).

Moreover, epidemiological studies have demonstrated a higher risk of asthma among overweight or obese children and adults (5–7). A previous study discovered that asthma incidence increased by a 2-fold risk in obese children (8). Of note, sex may modify the obesity–asthma relationship, with boys being at a higher risk of incident asthma than the risk in girls (8). A recent meta-analysis also reported an increased risk of asthma and wheeze associated with childhood obesity (6). However, the risk was more evident in girls than in boys (6). Furthermore, asthma may also contribute to the obesity epidemic. Two US cohort studies showed that children with asthma were at higher risk of developing obesity compared to those without asthma (9, 10). A pooled analysis of 16 European cohorts also suggested that children with early-life asthma may have an increased risk of incident obesity (11).

There is no systematic review addressing the association between asthma and risk of childhood obesity, although many systematic reviews have assessed the opposite direction (6–8). The bidirectional association of asthma and obesity may be established in a critical time window in early life. Although compelling evidence has demonstrated the co-occurrence of asthma and obesity in children and adolescents, it is still unclear whether one disorder contributes to the development of the other disorder and/or vice versa. Thus, the objective of this meta-analysis is to explore whether there was a possibility of a bidirectional association for these two disorders in children and adolescents, by assessing the association between obesity and asthma risk, as well as the association between asthma and obesity risk in children and adolescents.

## Materials and Methods

This meta-analysis was conducted in accordance with PRISMA statement (Supplementary Document 1) (12). The study characteristics are as follows: The study population should be children and adolescents. For the evaluation of the relationship between obesity and risk of asthma, the exposure should be obesity, and non-exposure should be normal weight. The outcome should be incident physician-diagnosed asthma. For the evaluation of the relationship between asthma and risk of obesity, the exposure should be asthma, and non-exposure should be non-asthma. The outcome should be incident obesity. Two researchers (L-SS and Q-LZ) independently performed the literature search, study screening and selection, and information extraction. Any discrepancies were resolved by discussion. A third reviewer (Y-XS) acted as an arbitrator whenever consensus was not reached.

### Literature Search and Study Selection

The PubMed, Embase, Web of Science, and CENTRAL databases were searched up to August 2020, using the Mesh terms and/or the combinations of the free words: (“obesity” or “body mass index” or “body weight”) and (“asthma”) and (“child” or “adolescent” or “children” or “adolescence” or “childhood”) and (“cohort” or “longitudinal” or “follow-up”). Google Scholar was also searched for gray literature. Reference lists of relevant studies were screened to identify other potential studies. The literature search was restricted to studies published in English. More details on the electronic search strategy can be obtained from Supplementary Document 2.

The inclusion criteria of this meta-analysis were as follows: (1) Studies should use a cohort design. (2) The study population should be children or adolescents. (3) For the evaluation of the association between obesity and asthma risk, the exposure of interest should be obesity, and outcome should be physician-diagnosed incident asthma; for the evaluation of the association between asthma and obesity risk, the exposure of interest should be physician-diagnosed asthma, and outcome should be incident obesity. (4) Studies should use the age- and sex-specific body mass index (BMI) as a measure of obesity in children (13). A child being obese was defined by BMI ≥ 95th percentile according to a children growth chart from the Centers for Disease Control and Prevention (14). (5) Physician-diagnosed asthma could be based on assessment from the medical records or clinical diagnosis, or self-reported with being diagnosed by a physician as having asthma. (6) Studies should provide risk estimates as risk ratio, rate ratio, hazard ratio, odds ratio, and the corresponding 95% confidence interval (CI) or standard error. When there were multiple risk estimates in a study, we used the one adjusted for most of the potential confounders.

The exclusion criteria were as follows: (1) not cohort studies, cross-sectional character of the analyses, or outcome of interest being present at baseline; (2) no obesity information; since the meta-analysis focused on the bidirectional association between obesity and asthma, studies that only reported risk information on other weight status or categories, such as per unit of BMI increase, overweight (85th ≤ BMI < 95th percentile), or overweight and obesity (BMI ≥ 85th percentile), were excluded from the review; (3) no information on physician-diagnosed asthma; studies that only reported information on wheezing or asthma medications were excluded from the review; and (4) no useful risk estimates. When more than one study was reported in the same study population, or newer data were available, we selected the most recent study.

### Information Extraction

A structured extraction form was used to collect information from the eligible studies. This form included details on the study author, year of publication, study location, participant source, study period, cohort size, number of cases, age at baseline, sex, exposure assessment, outcome ascertainment, risk estimates, and confounder adjustment.

### Quality Assessment

Two authors (L-SS and Q-LZ) independently evaluated the study quality using the Newcastle–Ottawa Scale (15). Where there was disagreement, a third reviewer (Y-XS) acted as an arbitrator. This tool was developed to evaluate the quality of non-randomized studies by using a star system in which a study was judged on three broad perspectives, including the selection of the study groups, the comparability of the groups, and the ascertainment of either the exposure or outcome of interest (15). This tool identified “high” quality choices with a star. Each numbered item within the “Selection” and “Outcome” categories can be given a maximum of one star (15). A maximum of two stars can be awarded for “Comparability.” More details on the Newcastle–Ottawa Scale could be obtained from Supplementary Document 3. There is no formal cut point to determine high-quality studies. According to the cut point used in the previous meta-analyses (16, 17), a study with a total score of ≥7 was considered as a high-quality study in this review.

### Statistical Analysis

We used the relative risk (RR) to measure the associations of obesity with risk of asthma, and asthma with risk of obesity. Heterogeneity was assessed by *I*^2^ statistic. A significant heterogeneity was considered at *I*^2^ > 50% (18). When there was significant between-study heterogeneity, the pooled RR was calculated using random-effects model (19); otherwise, fixed effects model was employed. Publication bias was assessed by funnel plot, as well as Begg's test and Egger's test (20, 21). All statistical analyses were performed using Stata 14.0 (Stata, College Station, TX, USA).

## Results

**Figure 1:**
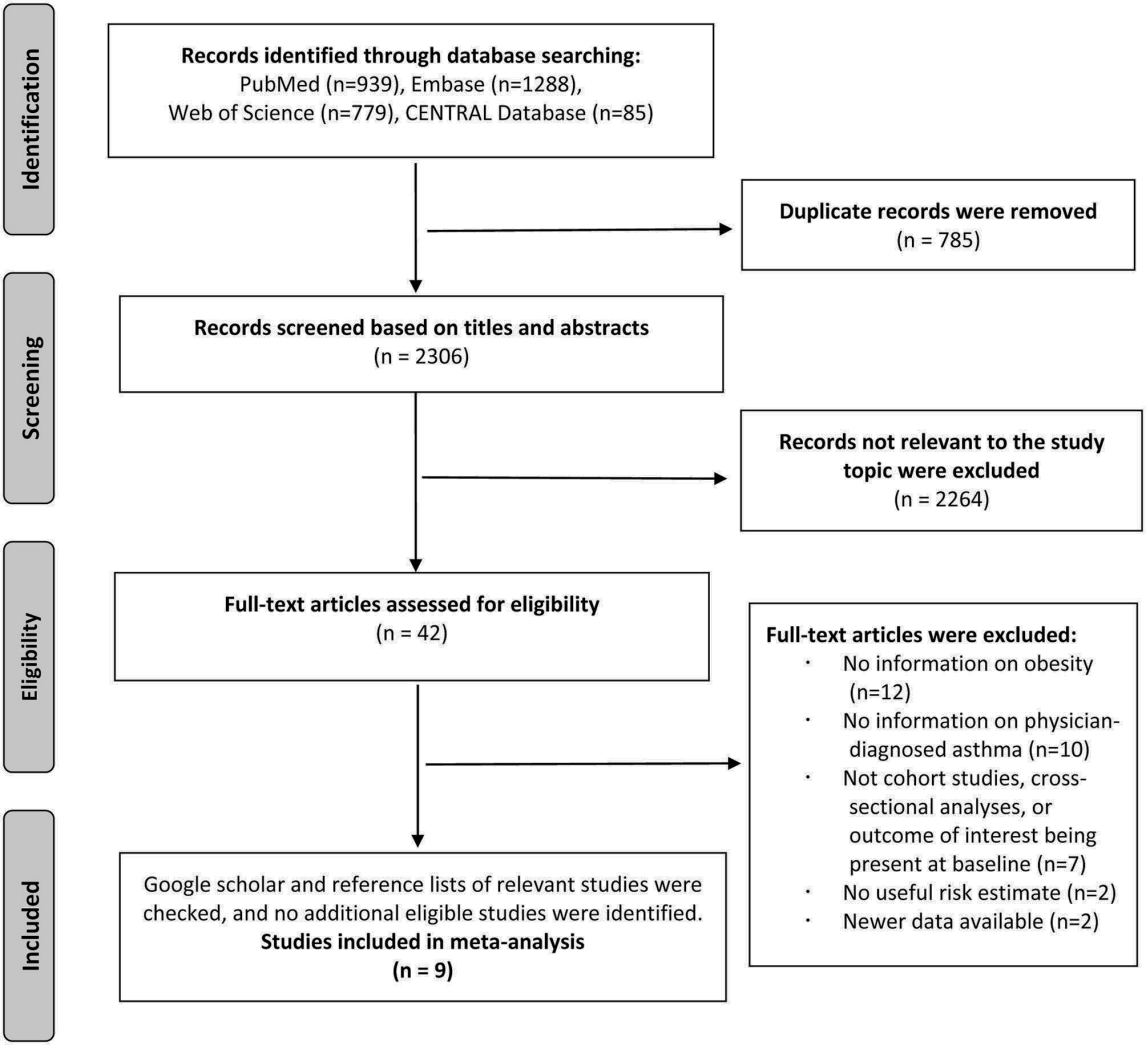
Flow chart of study selection.

The literature search identified 3,091 total records from PubMed, Embase, Web of Science, and CENTRAL databases, and 2,306 records remained after 785 duplicates were removed. A total of 2,264 records were excluded after screening the titles and abstracts. After reviewing the full texts of the remaining 42 studies, 12 studies were excluded because of not reporting information on obesity during childhood or adolescence; 10 studies were excluded because of not reporting information on the physician-diagnosed asthma; 7 studies were excluded because of not being cohort studies, cross-sectional character of the analyses, or the outcome being present at baseline; 2 studies were excluded because of no useful risk estimate; and 2 studies were excluded because of newer data available. The references of the excluded studies after the full-text review are listed in Supplementary Document 4. Thus, we included nine studies in the final analysis, of which six studies reported the association between obesity and risk of asthma (22–27) and three studies reported the association between asthma and risk of obesity (9–11) (Figure 1).

### Association Between Obesity and Risk of Asthma

For the obesity–asthma association, 1,112,758 children were included in the meta-analysis, with 74,066 children developing incident asthma. Three studies were conducted in the US, two studies in China, and one study in Sweden. For the obesity assessment at baseline, one study was conducted in children aged from 2 to 17 years old; one study was conducted in children at the age of 4 years old, and four studies were conducted in children aged more than 6 years old. For the physician-diagnosed asthma assessments, one study was conducted in children aged from 2 to 17 years, and other studies were conducted in children aged more than 7 years old. Studies by Ho et al. and Lee et al. had <3 years follow-up for outcomes to occur. More details of the characteristics of the included studies in the meta-analysis could be obtained from Supplementary Document 5. The definitions of asthma varied from study to study. Studies by Lang et al. and Black et al. assessed asthma status based on electronic health records, and four other studies assessed asthma status based on parents- or self-reported physician-diagnosed asthma. More details of the definitions of asthma are shown in Supplementary Document 6. In addition, all the studies were evaluated as high-quality studies, which indicated low risk of bias (Supplementary Document 7).

**Figure 2:**
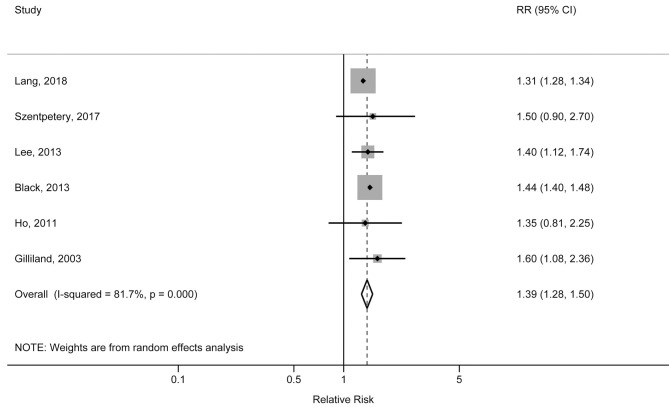
Forest plot of the association between obesity and risk of childhood asthma.

**Figure 3:**
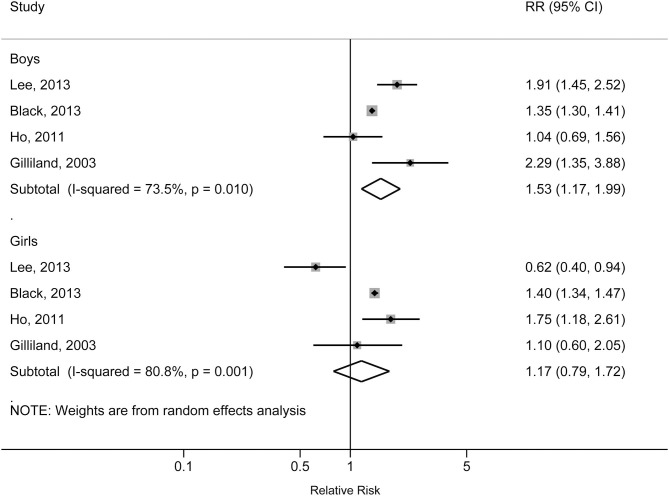
Forest plot of the association between obesity and risk of childhood asthma stratified by sex.

The individual studies included in the meta-analysis reported pooled RRs ranging from 1.31 (95% CI: 1.28, 1.34) for the Lang et al. study to 1.60 (95% CI: 1.08, 2.36) for the Gilliland et al. study. There was a statistically significant association between obesity and increased risk of physician-diagnosed asthma in children and adolescents. The pooled RR was 1.39 (95% CI: 1.28, 1.50; *p* < 0.001), with significant heterogeneity across studies (*I*^2^ = 81.7%, *p*_heterogeneity_ < 0.001) (Figure 2). The pooled RR for obesity with physician-diagnosed asthma risk in boys was 1.53 (95% CI: 1.17, 1.99; *p* = 0.002; *I*^2^ = 73.5%; *p*_heterogeneity_ = 0.010), but no significant association was observed in girls (RR = 1.17, 95% CI: 0.79, 1.72; *p* = 0.434; *I*^2^ = 80.8%; *p*_heterogeneity_ = 0.001) (Figure 3). When we excluded one study with preschool children included in the study population, the pooled RR for children at school age was 1.44 (95% CI: 1.40, 1.48; *p* < 0.001), and no statistical heterogeneity was detected (*I*^2^ = 0%, *p*_heterogeneity_ = 0.980). A visual inspection of the funnel plot and formally tested by Egger's test (*p* = 0.834) and Begg's test (*p* = 0.754) indicated no evidence of publication bias.

### Association Between Asthma and Risk of Obesity

For the three studies reporting asthma–obesity association, a total of 28,494 children with asthma were included in the meta-analysis, of which 1,261 children developed incident obesity during follow-up. Two studies were conducted in the US and one in European countries. For the asthma assessment at baseline, all studies were conducted in children under 8 years old. The ages at obesity assessment in the original studies varied from 5 to 18 years old. More details of the characteristics of the included studies in the meta-analysis could be obtained from Supplementary Document 5. The definitions of asthma varied from study to study; all studies assessed asthma status based on parent- or self-reported physician-diagnosed asthma. More details of the definitions of asthma are shown in Supplementary Document 6. All the studies were evaluated as high-quality studies, which indicated low risk of bias (Supplementary Document 7).

**Figure 4:**
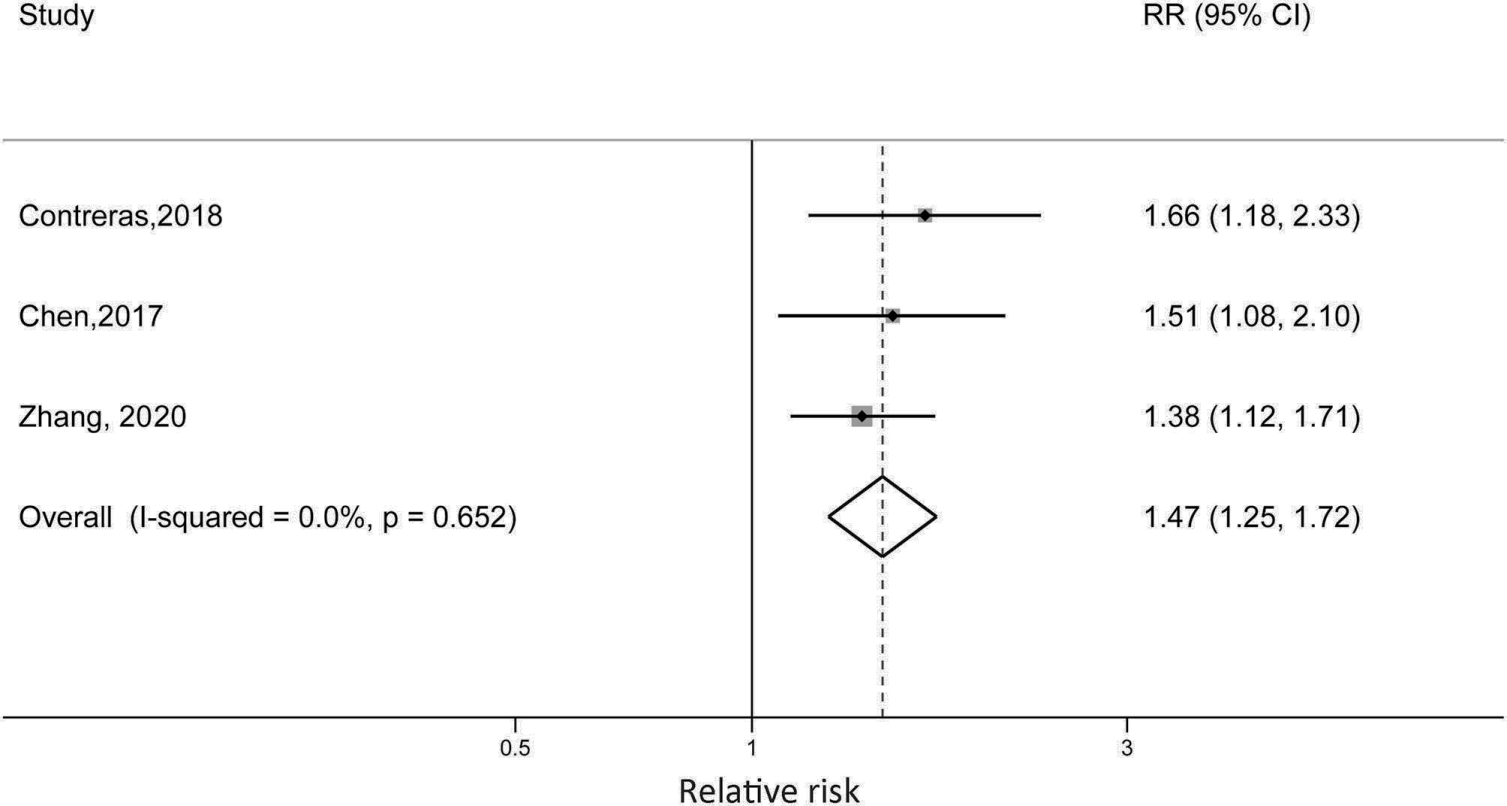
Forest plot of the association between early-onset asthma and risk of childhood obesity.

Zhang et al. reported that non-obese children with diagnosed asthma were at 38% increased risk of becoming obese compared with children without asthma (RR = 1.38; 95% CI: 1.12, 1.71). Contreras et al. reported an RR of 1.66 (95% CI: 1.18, 2.33) for European children, which was higher than the RR reported in the Chen et al. study (RR = 1.51, 95% CI: 1.08, 2.10). For the US cohort, the sex difference was detected. Boys with asthma at baseline were at 53% higher risk of developing obesity (RR = 1.53, 95% CI: 1.04, 2.26), while the reported RR in girls was 1.05 (95% CI: 0.54, 2.05). When we pooled the risk estimates from the three studies, the pooled RR was 1.47 (95% CI: 1.25, 1.72; *p* < 0.001) without statistical heterogeneity (*I*^2^ = 0%, *p*_heterogeneity_ = 0.652) (Figure 4).

## Discussion

To our knowledge, this is the first systematic review evaluating the association between asthma and risk of obesity in children and adolescents. In this meta-analysis, we found a bidirectional association between asthma and obesity during childhood and adolescence. Obese children were at an increased risk of developing physician-diagnosed asthma. Obese boys remained at significantly increased risk of asthma, but such a significant association was not observed in girls. Meanwhile, asthma was associated with a higher risk of developing obesity in children and adolescents.

Extensive epidemiological evidence assessed temporality of the two disorders and suggested that childhood obesity may be expected to either cause or worsen asthma (6, 28). Further studies also indicated that obesity duration may be associated with pulmonary function impairment in obese subjects (29, 30). It is worthy to note that not only the obese youth are more likely to develop asthma but also incident asthma may increase among children who are overweight (22, 25, 27). Furthermore, previous large cohort studies in the US and Australia suggested that an increase in BMI *z*-score and more rapid BMI gain during childhood may increase the risk of asthma in children and adolescents (31–34). The argument for causality is further supported by evidence from interventional trials that weight loss may improve the asthma-related outcomes (35).

Interestingly, sex-stratified subgroups showed that the association between obesity and asthma risk was significant among boys but not among girls in this meta-analysis. A previous study also reported that obese boys had higher risk than obese girls (boys: RR = 2.47; 95% CI, 1.57, 3.87; *p* < 0.001; girls: RR = 1.25; 95% CI, 0.51, 3.03; *p* = 0.63) (8). However, such was not the case in an updated meta-analysis that reported a more evident risk in girls (RR = 1.34, 95% CI: 1.16, 1.56; *p* < 0.001) than in boys (RR = 1.27, 95% CI: 1.15, 1.40; *p* < 0.001) (6). The prevalence of childhood asthma is higher in boys than in girls, while the prevalence of adult asthma is higher in women than in men. The reversal of sex difference may occur around puberty, indicating that sex hormones might play a role in the development of asthma (36). A previous study indicated an interaction between sex hormones and obesity on asthma development, but the exact mechanism was not well-known (37). The effects from the age may modify the gender difference in risk of developing asthma, considering that obese adolescents compared with school-aged children might have different risk factors for developing asthma such as hormonal factors (38). Further investigations on the effect of age on the gender difference of obesity–asthma association would be noteworthy. Moreover, sex may influence lung development and physiology starting at fetal lung developmental period (39). Surfactant production in the female neonate lung appears to be earlier than in male neonates, which may result in relatively lower airway resistance than male neonates (39, 40). Thus, preterm male fetuses, compared with females, have a higher prevalence of respiratory distress syndrome. In addition, compared with girls, boys have larger lungs but smaller airway diameters to their lung volume (39, 41). Thus, boys may be more vulnerable to later asthma than girls (40).

Although there is strong evidence that obesity may raise the risk of asthma, the present systematic review suggests that asthma may be also associated with a higher risk of developing obesity. Of note, sex difference was also observed for the association of asthma with risk of developing obesity in the US cohort (10). Boys with asthma at baseline were at higher risk of developing obesity than girls during follow-up (10). It is interesting that male sex may not only dominate moderating effects of obesity on asthma but also asthma on obesity development (10).

The development of these two childhood disorders may be a consequence of early-life alterations in the gut microbiome (42). Certain early life events such as mode of delivery, breastfeeding, and antibiotic use may alter the microbiome development and lead to microbiota disturbances (43). The complexity of early-life changes in gut microbiome such as delayed microbiome development and suppressed Clostridiales and Bacteroidetes populations may contribute to obesity and asthma (43, 44). Obesity may alter the gut bacterial community structure including production of bacterial-derived or modified metabolites, such as short-chain fatty acids (SCFAs) or bile acids, which play a role in the asthma development (45).

Obesity and asthma also share some etiological factors, such as a common genetic predisposition (46, 47). It is suggested that identified genes such as ACE, ADRB2, PRKCA, and TNF may influence both asthma and obesity simultaneously (46–48). Maternal smoking during pregnancy and utero exposures to ambient air polycyclic aromatic hydrocarbons have been reported as risk factors for both disorders during early life (49–51). Obesity and asthma may have common predisposing factors such as physical activity and diet (5). Moreover, children with asthma who used medication such as inhaled corticosteroids may increase the risk of obesity (4, 11). The development of asthma and obesity may be driven by different mechanisms. Further studies are still necessary to determine plausible mechanisms and understand how these mechanisms might interact with each other in the etiology of these two disorders.

A major strength of the meta-analysis is the large sample size, which could provide sufficient statistical power and greater reliability (precision) of the estimates. Moreover, we explored the temporal relationship of these two disorders in cohort studies, which may have relatively lower risk of presenting biases, such as reverse causation and recall bias, compared with other types of epidemiological designs (case-control or cross-section design) (52). Our study also has some limitations. First, there was significant heterogeneity among study results for the association between obesity and risk of asthma. To ensure the homogeneity of the included studies, we set relatively strict eligibility criteria. There are still several potential sources for the observed between-study heterogeneity. For example, the age of study population, socioeconomic status, and genetic diversity varied from study to study. For example, Lang et al. included children aged from 2 to 18 years (both preschool and school-aged children), but the other five studies only included the school-aged children with ages from 7 to 18 years. In the sensitivity analysis, when we excluded the Lang et al. study, the pooled RR of the remaining five studies slightly increased, but the statistical heterogeneity disappeared. Second, residual confounding inherent in the original studies may distort the associations. For example, the studies by Ho et al. and Black et al. did not adjust for comorbidities such as rhinitis, eczema, and food allergies in the multivariable models, which may lead to residual confounding. We conducted sensitivity analysis by excluding these two studies from the meta-analysis. The pooled RR of the remaining four studies, which considered the adjustment for the comorbidities in the analyses, did not change materially compared with the overall results (RR for sensitivity analysis = 1.31; 95% CI: 1.28, 1.34; *p* < 0.001). Third, the definitions of asthma varied from study to study. Physician assessment of asthma has been widely used as a method to assess the asthma status in epidemiological studies, but asthma diagnoses may be influenced by differences in clinical practice and heath care in different regions (53). We were not able to address these potential misclassifications inherent in the original studies. Fourth, since the BMI itself may not reflect where the fat is distributed in children, using the BMI itself to define obesity may therefore either under- or overestimate the obesity (14, 54). Fifth, the studies included in the meta-analysis are mainly from the European countries, US, and China, and little is known on the obesity–asthma association in the other areas such as Central and South America, Africa, and Oceania. Previous epidemiological studies from Australia, New Zealand, and Brazil suggested that a higher BMI may be associated with higher risks of asthma or wheezing in children and adolescents (31, 41, 55–59). These evidences may support the findings from the current meta-analysis; however, we should be cautious in generalizing the results of the meta-analysis to other areas, especially where the incidences of childhood asthma and obesity are relatively low. Last, although no publication bias was detected, the test for publication had low statistical power when small numbers of studies were included in the analysis. In addition, non-English studies were not searched and considered in this meta-analysis, which may lead to biased results.

This meta-analysis found a bidirectional association between obesity and asthma during childhood and adolescence, suggesting that obesity may increase the risk of childhood asthma, and asthma may also increase the risk of obesity. These findings shed light on the development of these early life disorders. Further studies may focus on investigating the potential mechanisms underlying these associations, as well as the systematic approaches for prevention of obesity and adequate treatments of asthma to reduce the trajectory toward these two diseases.

## Data Availability Statement

The data analyzed in this study was subject to the following licenses/restrictions: The dataset could be available from the corresponding author upon reasonable request. Requests to access these datasets should be directed to yunxiao.shang.sj.hospital@hotmail.com.

## Author Contributions

Y-XS developed the research design, interpreted the results, and also had primary responsibility for the final content. L-SS and Q-LZ analyzed the data and interpreted the results. L-SS, Q-LZ, and Y-XS drafted the manuscript. All authors critically reviewed and approved the manuscript.

## Conflict of Interest

The authors declare that the research was conducted in the absence of any commercial or financial relationships that could be construed as a potential conflict of interest.
