# Supplementary material for: Bidirectional Association Between Asthma and Obesity During Childhood and Adolescence: A Systematic Review and Meta-Analysis
Source: Front Pediatr. 2020 Oct 29;8:576858. doi: 10.3389/fped.2020.576858 (PMC7658650; doi:10.3389/fped.2020.576858)
Supplement: Supplementary file 2 [file Table_2.docx]

**Supplementary Document 2**

**Table 2** Search Strategy of the Meta-analysis

| **PubMed (n=939)** | ((((((obesity[MeSH Terms]) OR (body mass index[MeSH Terms])) OR (body weight[MeSH Terms])) AND (asthma[MeSH Terms])) AND ((child[MeSH Terms]) OR (adolescent[MeSH Terms]))) AND (((cohort studies[MeSH Terms]) OR (follow-up studies[MeSH Terms])) OR (longitudinal studies[MeSH Terms]))) OR (("obesity" OR "body mass index" OR "body weight") AND ("asthma") AND ("child" OR "adolescent" OR "children" OR "adolescence" OR "childhood") AND ("cohort" OR "longitudinal" OR "follow-up")) AND (english[Filter]) |
| --- | --- |
| **Embase (n=1288)** | (obesity OR 'body mass' OR 'body weight') AND asthma AND (child OR childhood OR adolescent OR adolescence) AND ('cohort analysis' OR 'longitudinal study' OR 'follow up') AND [english]/lim |
| **Web of Science (n=779)** | TOPIC: (Obesity OR "body mass index" OR "body weight") AND TOPIC: (asthma) AND TOPIC: (child OR children OR childhood OR adolescent OR adolescence) AND TOPIC: (cohort OR follow-up OR longitudinal) Refined by: LANGUAGES: (ENGLISH)  Timespan: All years.  Indexes: SCI-EXPANDED, CPCI-S, CCR-EXPANDED, IC. |
| **Central database**  **(1 review + 84 trials)** | obesity OR "body mass index" OR "body weight" in Title Abstract Keyword AND asthma in Title Abstract Keyword AND "child" OR "adolescent" OR "children" OR "adolescence" OR "childhood" in Title Abstract Keyword AND cohort OR longitudinal OR follow-up in Title Abstract Keyword - (Word variations have been searched) |
| **Google Scholar (n=374)** | allintitle: Obesity asthma Child OR Children OR Childhood OR adolescent OR adolescence |
