# Supplementary material for: Bidirectional Association Between Asthma and Obesity During Childhood and Adolescence: A Systematic Review and Meta-Analysis
Source: Front Pediatr. 2020 Oct 29;8:576858. doi: 10.3389/fped.2020.576858 (PMC7658650; doi:10.3389/fped.2020.576858)
Supplement: Supplementary file 3 [file Table_3.docx]

**Supplementary Document 3**

***Table 3*** *Newcastle-Ottawa Scale for cohort studies*

| **Selection**  1) Representativeness of the exposed cohort  a) truly representative of the average _______________ (describe) in the community **🟑**  b) somewhat representative of the average ______________ in the community **🟑**  c) selected group of users eg nurses, volunteers  d) no description of the derivation of the cohort  2) Selection of the non exposed cohort  a) drawn from the same community as the exposed cohort **🟑**  b) drawn from a different source  c) no description of the derivation of the non exposed cohort  3) Ascertainment of exposure  a) secure record (eg surgical records) **🟑**  b) structured interview **🟑**  c) written self report  d) no description  4) Demonstration that outcome of interest was not present at start of study  a) yes **🟑**  b) no |
| --- |
| **Comparability**  1) Comparability of cohorts on the basis of the design or analysis  a) study controls for _____________ (select the most important factor) **🟑**  b) study controls for any additional factor **🟑** (This criteria could be modified to indicate specific control for a second important factor.) |
| **Outcome**  1) Assessment of outcome  a) independent blind assessment **🟑**  b) record linkage **🟑**  c) self report  d) no description  2) Was follow-up long enough for outcomes to occur  a) yes (select an adequate follow up period for outcome of interest) **🟑**  b) no  3) Adequacy of follow up of cohorts  a) complete follow up - all subjects accounted for **🟑**  b) subjects lost to follow up unlikely to introduce bias - small number lost - > ____ % (select an adequate %) follow up, or description provided of those lost) **🟑**  c) follow up rate < ____% (select an adequate %) and no description of those lost  d) no statement |
| **CODING MANUAL FOR COHORT STUDIES**  ***SELECTION***   1. **Representativeness of the Exposed Cohort**   Item is assessing the representativeness of exposed individuals in the community, not the representativeness of the sample of women from some general population. For example, subjects derived from groups likely to contain middle class, better educated, health oriented women are likely to be representative of postmenopausal estrogen users while they are not representative of all women (e.g. members of a health maintenance organisation (HMO) will be a representative sample of estrogen users. While the HMO may have an under-representation of ethnic groups, the poor, and poorly educated, these excluded groups are not the predominant users users of estrogen).  Allocation of stars as per rating sheet   1. **Selection of the Non-Exposed Cohort**   Allocation of stars as per rating sheet   1. **Ascertainment of Exposure**   Allocation of stars as per rating sheet   1. **Demonstration That Outcome of Interest Was Not Present at Start of Study**   In the case of mortality studies, outcome of interest is still the presence of a disease/ incident, rather than death. That is to say that a statement of no history of disease or incident earns a star.  ***COMPARABILITY***   1. **Comparability of Cohorts on the Basis of the Design or Analysis**   A maximum of 2 stars can be allotted in this category  Either exposed and non-exposed individuals must be matched in the design and/or confounders must be adjusted for in the analysis. Statements of no differences between groups or that differences were not statistically significant are not sufficient for establishing comparability. Note: If the relative risk for the exposure of interest is adjusted for the confounders listed, then the groups will be considered to be comparable on each variable used in the adjustment.  There may be multiple ratings for this item for different categories of exposure (e.g. ever vs. never, current vs. previous or never)  Age = , Other controlled factors =  ***OUTCOME***   1. **Assessment of Outcome**   For some outcomes (e.g. fractured hip), reference to the medical record is sufficient to satisfy the requirement for confirmation of the fracture. This would not be adequate for vertebral fracture outcomes where reference to x-rays would be required.   1. Independent or blind assessment stated in the paper, or confirmation of the outcome by reference to secure records (x-rays, medical records, etc.) 2. Record linkage (e.g. identified through ICD codes on database records) 3. Self-report (i.e. no reference to original medical records or x-rays to confirm the outcome) 4. No description. 5. **Was Follow-Up Long Enough for Outcomes to Occur**   An acceptable length of time should be decided before quality assessment begins (e.g. 5 yrs. for exposure to breast implants)   1. **Adequacy of Follow Up of Cohorts**   This item assesses the follow-up of the exposed and non-exposed cohorts to ensure that losses are not related to either the exposure or the outcome.  Allocation of stars as per rating sheet |

Note: A study can be awarded a maximum of one star for each numbered item within the Selection and Outcome categories. A maximum of two stars can be given for Comparability
