# Supplementary material for: Bidirectional Association Between Asthma and Obesity During Childhood and Adolescence: A Systematic Review and Meta-Analysis
Source: Front Pediatr. 2020 Oct 29;8:576858. doi: 10.3389/fped.2020.576858 (PMC7658650; doi:10.3389/fped.2020.576858)
Supplement: Supplementary file 4 [file Table_4.docx]

**Supplementary Document 4**

***Table 4 Excluded studies after full-text review***

| 1. Twelve studies were excluded because of not reporting information on obesity during childhood or adolescence (1-12); 2. Ten studies were excluded because of not reporting information on the physician-diagnosed asthma (13-22); 3. Seven studies were excluded because of the outcome being present at baseline, cross-sectional character of the analyses, or not prospective studies (23-29); 4. Two studies were excluded because of no useful risk estimate (30, 31); 5. Two study were excluded because of newer data available in the same study population (32, 33). |
| --- |
| **References**  1. Casas M, Den Dekker HT, Kruithof CJ, Reiss IK, Vrijheid M, Sunyer J, et al. The effect of early growth patterns and lung function on the development of childhood asthma: A population based study. Thorax. 2018;73(12):1137-45.  2. Tsai HJ, Wang G, Hong X, Yao TC, Ji Y, Radovick S, et al. Early life weight gain and development of childhood asthma in a prospective birth cohort. Ann Am Thorac Soc. 2018;15(10):1197-204.  3. Belfort MB, Cohen RT, Rhein LM, McCormick MC. Preterm infant growth and asthma at age 8 years. Arch Dis Child Fetal Neonatal Ed. 2016;101(3):F230-F4.  4. Lawson JA, Janssen I, Bruner MW, Hossain A, Pickett W. Asthma incidence and risk factors in a national longitudinal sample of adolescent Canadians: a prospective cohort study. BMC Pulm Med. 2014;14:51.  5. Granell R, Henderson AJ, Evans DM, Smith GD, Ness AR, Lewis S, et al. Effects of BMI, fat mass, and lean mass on asthma in childhood: a Mendelian randomization study. PLoS Med. 2014;11(7):e1001669.  6. Zhang Z, Lai HJ, Roberg KA, Gangnon RE, Evans MD, Anderson EL, et al. Early childhood weight status in relation to asthma development in high-risk children. J Allergy Clin Immunol. 2010;126(6):1157-62.  7. Mamun AA, Lawlor DA, Alati R, O'Callaghan MJ, Williams GM, Najman JM. Increasing body mass index from age 5 to 14 years predicts asthma among adolescents: Evidence from a birth cohort study. Int J Obes (Lond). 2007;31(4):578-83.  8. Mannino DM, Mott J, Ferdinands JM, Camargo Jr CA, Friedman M, Greves HM, et al. Boys with high body masses have an increased risk of developing asthma: Findings from the National Longitudinal Survey of Youth (NLSY). Int J Obes (Lond). 2006;30(1):6-13.  9. Gold DR, Damokosh AI, Dockery DW, Berkey CS. Body-mass index as a predictor of incident asthma in a prospective cohort of children. Pediatr Pulmonol. 2003;36(6):514-21.  10. Porter M, Wegienka G, Havstad S, Nageotte CG, Johnson CC, Ownby DR, et al. Relationship between childhood body mass index and young adult asthma. Annals of Allergy, Asthma and Immunology. 2012;109(6):408-11.  11. Chastang J, Baiz N, Parnet L, Cadwallader JS, De Blay F, Caillaud D, et al. Changes in body mass index during childhood and risk of various asthma phenotypes: a retrospective analysis. Pediatric Allergy and Immunology. 2017;28(3):273-9.  12. Rzehak P, Wijga AH, Keil T, Eller E, Bindslev-Jensen C, Smit HA, et al. Body mass index trajectory classes and incident asthma in childhood: results from 8 European Birth Cohorts--a Global Allergy and Asthma European Network initiative. J Allergy Clin Immunol. 2013;131(6):1528-36.  13. Chinn S, Rona RJ. Can the increase in body mass index explain the rising trend in asthma in children? Thorax. 2001;56(11):845-50.  14. Magnusson JÖ, Kull I, Mai XM, Wickman M, Bergström A. Early childhood overweight and asthma and allergic sensitization at 8 years of age. Pediatrics. 2012;129(1):70-6.  15. Castro-Rodriguez JA, Holberg CJ, Morgan WJ, Wright AL, Martinez FD. Importance of acute Mycoplasma pneumoniae and Chlamydia pneumoniae infections in children with wheezing. Am J Respir Crit Care Med. 2001;163(6):1344-9. .  16. McCallister M, Medrano R, Wojcicki J. Early life obesity increases the risk for asthma in San Francisco born Latina girls. Allergy Asthma Proc. 2018;39(4):273-80.  17. Mandhane PJ, Greene JM, Cowan JO, Taylor DR, Sears MR. Sex differences in factors associated with childhood- and adolescent-onset wheeze. Am J Respir Crit Care Med. 2005;172(1):45-54.  18. Noal RB, Menezes AMB, MacEdo SEC, Dumith SC, Perez-Padilla R, Araújo CLP, et al. Is obesity a risk factor for wheezing among adolescents? A prospective study in Southern Brazil. J Adolesc Health. 2012;51(SUPPL. 6):S38-S45.  19. Scholtens S, Wijga AH, Seidell JC, Brunekreef B, de Jongste JC, Gehring U, et al. Overweight and changes in weight status during childhood in relation to asthma symptoms at 8 years of age. J Allergy Clin Immunol. 2009;123(6):1312-8.e2.  20. Taveras EM, Rifas-Shiman SL, Camargo Jr CA, Gold DR, Litonjua AA, Oken E, et al. Higher adiposity in infancy associated with recurrent wheeze in a prospective cohort of children. J Allergy Clin Immunol. 2008;121(5):1161-6.e3.  21. Tollefsen E, Langhammer A, Romundstad P, Bjermer L, Johnsen R, Holmen TL. Female gender is associated with higher incidence and more stable respiratory symptoms during adolescence. Respir Med. 2007;101(5):896-902.  22. Wake M, Canterford L, Patton GC, Hesketh K, Hardy P, Williams J, et al. Comorbidities of overweight/obesity experienced in adolescence: Longitudinal study. Arch Dis Child. 2010;95(3):162-8.  23. Lu FL, Hsieh CJ, Caffrey JL, Lin MH, Lin YS, Lin CC, et al. Body mass index may modify asthma prevalence among low-birth-weight children. Am J Epidemiol. 2012;176(1):32-42.  24. Mai XM, Gäddlin PO, Nilsson L, Leijon I. Early rapid weight gain and current overweight in relation to asthma in adolescents born with very low birth weight. Pediatr Allergy Immunol. 2005;16(5):380-5.  25. Loid P, Goksör E, Alm B, Pettersson R, Möllborg P, Erdes L, et al. A persistently high body mass index increases the risk of atopic asthma at school age. Acta Paediatr. 2015;104(7):707-12.  26. Sidoroff V, Hyvärinen MK, Piippo-Savolainen E, Korppi M. Overweight does not increase asthma risk but may decrease allergy risk at school age after infantile bronchiolitis. Acta Paediatr. 2012;101(1):43-7.  27. Jartti T, Saarikoski L, Jartti L, Lisinen I, Jula A, Huupponen R, et al. Obesity, adipokines and asthma. Allergy. 2009;64(5):770-7.  28. Bibi H, Shoseyov D, Feigenbaum D, Genis M, Friger M, Peled R, et al. The relationship between asthma and obesity in children: Is it real or a case of over diagnosis? Journal of Asthma. 2004;41(4):403-10.  29. von Mutius E, Schwartz J, Neas LM, Dockery D, Weiss ST. Relation of body mass index to asthma and atopy in children: the National Health and Nutrition Examination Study III. Thorax. 2001;56(11):835-8.  30. Green TL. Examining the temporal relationships between childhood obesity and asthma. Econ Hum Biol. 2014;14:92-102.  31. Oddy WH, Sherriff JL, De Klerk NH, Kendall GE, Sly PD, Beilin LJ, et al. The relation of breastfeeding and body mass index to asthma and atopy in children: A prospective cohort study to age 6 years. American Journal of Public Health. 2004;94(9):1531-7.  32. Zhang Y, Chen Z, Berhane K, Urman R, Chatzi V, Breton C, et al. Dynamic relationship between asthma and obesity in the CHS: Insights from joint transition models. American Journal of Respiratory and Critical Care Medicine. 2018;197(Meeting Abstracts).  33. Lang J, Hossain M, Wysocki T, Bunnell T. Effect of obesity on incident childhood asthma: Results from the national pedsnet clinical data research network. American Journal of Respiratory and Critical Care Medicine. 2018;197(Meeting Abstracts). |
