# Supplementary material for: Bidirectional Association Between Asthma and Obesity During Childhood and Adolescence: A Systematic Review and Meta-Analysis
Source: Front Pediatr. 2020 Oct 29;8:576858. doi: 10.3389/fped.2020.576858 (PMC7658650; doi:10.3389/fped.2020.576858)
Supplement: Supplementary file 5 [file Table_5.docx]

**Supplementary Document 5**

***Table 5 Characteristics of the included studies***

| ***Obesity and risk of childhood asthma*** | | | | | | | | | |
| --- | --- | --- | --- | --- | --- | --- | --- | --- | --- |
| **Study** | **Location** | **Cohort size** | **Number of Cases** | **Sex** | **Study period** | **Obesity assessment** | **Asthma assessment** | **Risk estimate and 95%CI** | **Confounder Adjustments** |
| Lang, 2018 | US | 507496 | 41330 | Male: 50.73% | 2009-2015; mean follow-up is 4 years. | BMI in the ≥95th percentile adjusted for age and sex at age of 2 to 17 years. | Asthma assessment based on electronic health records. The incidence of asthma during the observation period (age at 2 to 17 years), defined as ≥2 encounters with a diagnosis of asthma and ≥1 asthma medication prescription. | 1.31 (95%CI: 1.28, 1.34) | Age, race, ethnicity, sex, site, insurance status, baseline allergic rhinitis, baseline food allergy, and baseline proton pump inhibitor use. |
| Szentpetery, 2017 | Sweden | 2290 | 121 | Male: 49.87% | 12 years | BMI z-score ≥95th percentile at age of 4 years | Parents-reported physician-diagnosed asthma at ages of 12 years | 1.50 (95%CI: 0.90, 2.70) | Sex, parental asthma, allergic rhinitis at age 4 years and early-life second hand smoke |
| Lee, 2013 | Taiwan, China | 3,547 | 50 | Male: 51.58% | 2007-2009 | Sex and age-normalized BMI ≥95th percentile at 12 to 13 years old at study entry | Parents-reported physician-diagnosed asthma; Diagnosis of asthma over the 2-year period of follow-up at age of 14-15 years | Both: 1.40 (95%CI: 1.12, 1.74);  Boys: 1.91 (95%CI: 1.45, 2.52);  Girls: 0.62 (95%CI: 0.40, 0.94) | Sex, age, community, in-utero exposure to maternal smoking, current SHS, allergic rhinitis and atopic eczema |
| Black, 2013 | US | 591581 | 31,777 | Male: 50.87% | 2007-2011; The median length of follow-up was 3 years (interquartile range,  1.7–4.0 years) | Moderately obese defined as ≥95th percentile or BMI ≥30, and extremely obese defined as ≥1.2 × 95th percentile or BMI ≥35. BMI was assessed at least 6 years of age at the time of the first measurement (baseline) | Incident asthma based on electronic health record. Asthma was assessed at age of <19 years. | Both: 1.44 (95%CI: 1.40, 1.48);  Boys: 1.35 (95%CI: 1.30, 1.41);  Girls: 1.40 (95%CI: 1.34, 1.47) | Age, sex, race/ethnicity, and payer for insurance |
| Ho, 2011 | Taiwan, China | 4052 | 500 | Male: 54.4% | 1995-1996; 12-month follow-up | BMI was calculated and classified into under-weight, normal, overweight and obese, using age- and sex-specific thresholds at 13-15 years | Physician-diagnosed asthma was based on uniform criteria that included the New England core questionnaire, the ISAAC video questionnaire at 12 months later at age of 14-16 years | Both: 1.35 (95%CI: 0.81, 2.25);  Boys: 1.04 (95%CI: 0.69, 1.56);  Girls: 1.75 (95%CI: 1.18, 2.61) | Breastfeeding, fungus/mould at home, parental history of asthma, and parent education along with age, exercise frequency, air condition usage, cigarette smoking, environmental tobacco  smoke, and pet ownership |
| Gilliland, 2003 | US | 3792 | 288 | Male: 47.44% | 1993-1998; 4-year period of follow-up | BMI > 95th% at age of 7–18 years | Self-reported physician-diagnosed asthma at ages of 7-18 years. Children were assessed annually during school visits until high school graduation | Both: 1.60 (95%CI: 1.08, 2.36);  Boys: 2.29 (95%CI: 1.35, 1.99)；  Girls: 1.10 (95%CI: 0.60, 1.72) | Age, sex, race, health insurance, community, parental history of asthma/allergies, birth weight, humidifier use, wheeze, allergy, team sports participation, smoking, household environmental tobacco  smoke, household pets and pests, puberty, and lung function |
| ***Asthma and risk of childhood obesity*** | | | | | | | | | |
| **Study** | **Location** | **Cohort size** | **Number of Cases** | **Sex** | **Study period** | **Asthma assessment** | **Obesity assessment** | **Risk estimate and 95% CI** | **Confounder Adjustments** |
| Zhang, 2020 | US | 5193 | 436 | 2658 boys and 2535 girls | 2003-2012; up to 10 years of follow-up | Asthma status was ascertained by self-reported physician-diagnosed asthma. At the time of asthma assessment, the children are kindergarten and first-grade students, and 62.7% and 37.3% children with ages of <7 years and ≥ 7 years respectively. | Obesity was defined as BMI at or above 95th  percentile for children based on the age- and sex-specific BMI growth curve from the US Centers for Disease Control and Prevention. Children obesity status was assessed during 10 years of follow-up up to the age of 18 years old. | 1.38 (95%CI: 1.12, 1.71) | Age, sex, race/ethnicity, parent’s education, annual family income, child’s insurance status, exposure to maternal smoking in utero and environmental tobacco smoke, Spanish language questionnaire indicator, the community of residence, physical activity and medication status. |
| Contreras, 2018 | European countries | 21130 | 483 obesity children | 10791 boys and 10339 girls | 1990-2008  non-obese children at 3–4 years of age were followed for incident obesity up to 8 years of age. | Asthma status was ascertained by patents-reported physician-diagnosed asthma. Physician-diagnosed asthma, wheezing and allergic rhinitis were assessed up to 3-4 years of age. Information on asthma, wheeze and allergic rhinitis in the participating cohorts was obtained from questionnaires that were adapted from the International Study on Asthma and Allergy in Childhood (ISAAC) and administered to parents. | Weight and height information based on clinical examinations, health records, or parental-reported questionnaires at 3–4 years of age and at least one other time point between 5–8 years of age. Obese status was defined according to the 2012 Cole-International Obesity Task Force age and sex-specific cut-offs. | 1.66 (95%CI: 1.18, 2.33) | Cohort, age at baseline, smoking during pregnancy, passive smoke, parity, maternal education, parental asthma, breastfeeding and birthweight with a sex-specific baseline hazard. |
| Chen, 2017 | US | 2171 | 342 obesity children | 1087 boys and 1084 girls | 2002-2012 The median length  of follow-up was 6.9 years | Children asthma status was ascertained by parent- or self-reported physician-diagnosed asthma at 5-8 years of age at study enrollment | Height and weight were measured annually, and the mean age is 15.2 years at last assessment. Obese categories were determined using the 95th BMI percentile thresholds based on the age and sex-specific Center for Disease Control 2000 BMI growth curves. | Both: 1.51 (95%CI: 1.08, 2.10);  Boys: 1.53, 95%CI: 1.04, 2.26).  Girls: 1.05 (95% CI: 0.54, 2.05) | The community, age, ethnicity, annual family income, parental education levels, children’s health insurance coverage (yes/no), number of exercise classes attended in the previous year, weekly days of outdoor sports, overweight status (yes/no), second-hand smoke and maternal smoking exposure in utero., and follow-up time-dependent variables including number of exercise classes attended, weekly days of outdoor sports, and any asthma medication use with a sex-specific baseline hazard. |

BMI, body mass index; CI, confidence interval
