# Supplementary material for: Bidirectional Association Between Asthma and Obesity During Childhood and Adolescence: A Systematic Review and Meta-Analysis
Source: Front Pediatr. 2020 Oct 29;8:576858. doi: 10.3389/fped.2020.576858 (PMC7658650; doi:10.3389/fped.2020.576858)
Supplement: Supplementary file 6 [file Table_6.docx]

**Supplementary Document 6**

**Table 6** Definition of asthma in the original studies

| **Study** | **Definition of asthma** |
| --- | --- |
| ***Association between obesity and asthma risk*** | |
| Lang, 2018 | **Asthma assessment based on electronic health records.**  The incidence of asthma during the observation period (age at 2 to 17 years), defined as ≥2 encounters with a diagnosis of asthma and ≥1 asthma medication prescription.  Secondary outcomes included looser and stricter criteria for an asthma diagnosis, including ≥1 encounter with an asthma diagnosis, ≥2 encounters with an asthma diagnosis (without the requirement of an asthma drug prescription), and ≥2 encounters with an asthma diagnosis with an additional confirmation by spirometry. |
| Szentpetery, 2017 | **Parents-reported physician-diagnosed asthma** at ages of 12 years.  Asthma defined as physician-diagnosed asthma and at least one episode of wheeze in the prior year. |
| Lee, 2013 | **Parents-reported physician-diagnosed asthma;** Diagnosis of asthma over the 2-year period of follow-up at age of 14-15 years.  The incident asthma was defined by the question ‘‘Has a physician ever diagnosed your child as having asthma?’’ on baseline and follow-up questionnaire. |
| Black, 2013 | **Asthma assessment based on electronic health records.** Asthma was assessed at age of <19 years.  Incident asthma cases were identified by any of the following 3 conditions: 1) the presence of a physician diagnosis of asthma identified by the ICD-9, code 493 associated with any medical encounter and at least 1 pharmacy dispensing of an asthma-specific medication in the same year, 2) the presence of 1 or more asthma-related emergency department visits or hospitalizations in the absence of asthma medication, or 3) 3 or more asthma-related ambulatory visits in the absence of asthma medication. All other patients, including those who had 2 or fewer ambulatory visits accompanied by an asthma diagnosis but no asthma medications, as well as those who had asthma medications but no asthma diagnosis in the electronic health record, were classified as not having asthma. |
| Ho, 2011 | **Parents- or self-reported physician-diagnosed asthma** at ages of 14-16 years.  Physician-diagnosed asthma was based on uniform criteria that included the New England core questionnaire, the ISAAC video questionnaire, based on both parent and student answers guided by adolescent respiratory system technical consultants, and ATS-PFT standards. Percent predicted PFT values were computed for each individual adjusted for age, gender, and height. The reversibility criteria used in the present study were determined as FEV1/FVC < 70 along with the percent change in FEV1. |
| Gilliland, 2003 | **Self-reported physician-diagnosed asthma** at ages of 7-18 years. Children were assessed annually during school visits until high school graduation.  An incident asthma case was defined as a new physician diagnosis of asthma during the time between follow-up assessments. |
| ***Association between asthma and obesity risk*** | |
| Zhang, 2019 | **Parents- or self-reported physician-diagnosed asthma;** At the time of asthma assessment, the children are kindergarten and first-grade students, and 62.7% and 37.3% children with ages of <7 years and ≥ 7 years respectively.  Asthma was defined based on answers in questionnaires. When a parent or legal guardian answered ‘‘yes’’ to the question ‘‘Has a doctor diagnosed your child with asthma?’’ in the baseline questionnaire, or the child answered ‘‘yes’’ to the question ‘‘Has a doctor ever said you had asthma?’’ in the annual questionnaires during study follow-up, the child was classified from then on as having asthma. |
| Contreras, 2018 | **Parents-reported physician-diagnosed asthma** at any point up to 3-4 years of age.  Information on asthma, wheeze and allergic rhinitis in the participating cohorts was obtained from questionnaires that were adapted from the International Study on Asthma and Allergy in Childhood (ISAAC) and administered to parents. Presence of asthma at baseline (yes/no) was determined by report of physician diagnosis of asthma. For asthma-related phenotypes, this study defined asthma history based on responses to the baseline asthma and wheeze questions and categorized as: 1) active asthma (have baseline asthma and baseline wheeze), 2) have baseline asthma, but no baseline wheeze, 3) have no baseline asthma, but have baseline wheeze, and 4) have no baseline asthma and no baseline wheeze (reference category). |
| Chen, 2017 | **Parents-reported physician-diagnosed asthma** at age of 5-8 years.  Asthma history was classified based on a yes/no response to the question “Has a doctor ever diagnosed this child as having asthma”? Active asthma was defined as children with lifetime asthma and wheeze during the  previous year of the study visit. History of asthma medication use was assessed based on questions about any rescue, controller, and other medication use for asthma or wheezing in the last 12 months.  The age of asthma onset was classified into “early” (<4 year of age) and “late” (older than 4 year of age). |
