# Supplementary material for: Bidirectional Association Between Asthma and Obesity During Childhood and Adolescence: A Systematic Review and Meta-Analysis
Source: Front Pediatr. 2020 Oct 29;8:576858. doi: 10.3389/fped.2020.576858 (PMC7658650; doi:10.3389/fped.2020.576858)
Supplement: Supplementary file 7 [file Table_7.docx]

**Supplementary Document 7**

**Table 7** Study quality of cohort studies included in the meta-analysis *

| **Study** | **Selection** | | | | **Comparability** | **Outcome** | | | **Total score** |
| --- | --- | --- | --- | --- | --- | --- | --- | --- | --- |
|  | **Representativeness of the exposed cohort** | **Selection of the unexposed**  **cohort** | **Ascertainment**  **of exposure** | **Outcome of interest not present at start of study** | **Control for**  **potential confounders†** | **Assessment of outcome** | **Follow-up**  **long enough for outcomes**  **to occur ‡** | **Adequacy of**  **follow-up**  **of cohorts §** |  |
| Zhang, 2020 | ⚝ | ⚝ | ⚝ | ⚝ | ⚝⚝ | ⚝ | ⚝ | ⚝ | 9 |
| Contreras, 2018 | ⚝ | ⚝ | ⚝ | ⚝ | ⚝⚝ | ⚝ | ⚝ | ⚝ | 9 |
| Lang, 2018 | ⚝ | ⚝ | ⚝ | ⚝ | ⚝⚝ | ⚝ | ⚝ | ⚝ | 9 |
| Chen, 2017 | ⚝ | ⚝ | ⚝ | ⚝ | ⚝⚝ | ⚝ | ⚝ | ⚝ | 9 |
| Szentpetery, 2017 | ⚝ | ⚝ | ⚝ | ⚝ | ⚝⚝ | ⚝ | ⚝ | ⚝ | 9 |
| Lee, 2013 | ⚝ | ⚝ | ⚝ | ⚝ | ⚝⚝ | ⚝ | - | ⚝ | 8 |
| Black, 2013 | ⚝ | ⚝ | ⚝ | ⚝ | ⚝ | ⚝ | ⚝ | ⚝ | 8 |
| Ho, 2011 | - | ⚝ | ⚝ | ⚝ | ⚝⚝ | ⚝ | - | ⚝ | 7 |
| Gilliland, 2003 | ⚝ | ⚝ | ⚝ | ⚝ | ⚝⚝ | ⚝ | ⚝ | ⚝ | 9 |

† A maximum of 2 stars could be awarded for this item. Studies that controlled for age and sex received one star, whereas studies that controlled for most of the other important confounders such as baseline allergic rhinitis, baseline food allergy, exposure to maternal smoking in utero, environmental tobacco smoke, physical activity or medication status received an additional star.

‡ A cohort study with a follow-up time >3 years was assigned one star.

§ A cohort study with subjects lost to follow up unlikely to introduce bias, or a follow-up rate >80% was assigned one star.
